# Scaling in ANOVA-simultaneous component analysis

**DOI:** 10.1007/s11306-015-0785-8

**Published:** 2015-02-14

**Authors:** Marieke E. Timmerman, Huub C. J. Hoefsloot, Age K. Smilde, Eva Ceulemans

**Affiliations:** 1 University of Groningen, Grote Kruisstraat 2/1, 9712TS Groningen, The Netherlands; 2 Biosystems Data Analysis, Faculty of Sciences, University of Amsterdam, Amsterdam, The Netherlands; 3 KU Leuven, Leuven, Belgium

**Keywords:** Pre-treatment, Designed experiments, High-dimensional data

## Abstract

In omics research often high-dimensional data is collected according to an experimental design. Typically, the manipulations involved yield differential effects on subsets of variables. An effective approach to identify those effects is ANOVA-simultaneous component analysis (ASCA), which combines analysis of variance with principal component analysis. So far, pre-treatment in ASCA received hardly any attention, whereas its effects can be huge. In this paper, we describe various strategies for scaling, and identify a rational approach. We present the approaches in matrix algebra terms and illustrate them with an insightful simulated example. We show that scaling directly influences which data aspects are stressed in the analysis, and hence become apparent in the solution. Therefore, the cornerstone for proper scaling is to use a scaling factor that is free from the effect of interest. This implies that proper scaling depends on the effect(s) of interest, and that different types of scaling may be proper for the different effect matrices. We illustrate that different scaling approaches can greatly affect the ASCA interpretation with a real-life example from nutritional research. The principle that scaling factors should be free from the effect of interest generalizes to other statistical methods that involve scaling, as classification methods.

**Electronic supplementary material:**

The online version of this article (doi:10.1007/s11306-015-0785-8) contains supplementary material, which is available to authorized users.

## Introduction

In metabolomics, transcriptomics, proteomics and next generation sequencing (NGS), experimental data are obtained on the abundance of (very) large numbers of biomolecules in biological material. Typically, the manipulations involved yield differential effects on subsets of such biomolecules since they are often obtained under different conditions (treatment, time,…). In the absence of specific theoretical guidance, the key challenge is to unravel the nature of the differential effects and the associated subsets of biomolecules on the basis of the empirical data. We will focus on metabolomics, but the methods described also translate to other (omics) data.

A classical and powerful tool to identify such subsets is principal component analysis (PCA). PCA reveals dominant sources of variance in the observed data. However, straightforward use of PCA is typically not effective in this setting, because the thus identified dominant sources will not necessarily be linked to the experimental manipulations. In particular the between-subject variability within experimental conditions is often large (Xu et al. 2014), implying that the effects of the manipulations remain hidden in PCA results.

An effective exploratory alternative that accounts for the experimental design, and hence explicitly identifies and disentangles sources of variation due to the experimental manipulations, is ANOVA-simultaneous component analysis (ASCA) (Jansen et al. 2005; Smilde et al. 2005). The core idea of ASCA is to decompose the observed data matrix into a series of additive effect matrices, according to the experimental design, and subsequently perform a PCA on the effect matrices of interest. The latter is done to identify the dominant sources of variation for that particular effect. The ASCA approach has been fruitfully applied in a range of application areas (de Noord and Theobald 2005; Ferreira et al. 2009; Xu et al. 2014).

Although ASCA sheds light on different sources of variation, its results may still heavily depend on differences in variances of the metabolites within each of the effect matrices and thus depend on the pre-treatment applied to data. Pre-treatment is standard practice in metabolomics in order to focus on the biologically relevant information (Goodacre et al. 2007; van den Berg et al. 2006).

The pre-treatment steps in ASCA are the same ones as in PCA: centering, typically around the mean, and scaling. Scaling pertains to dividing each variable by a factor, for example the variable’s standard deviation. The possibly large effects of the specific pre-treatment applied are well-known in the context of PCA (e.g., Vandenginste et al. 1998) but received hardly any attention in ASCA so far. This lack of attention is understandable for centering, because this type of pre-treatment is implicitly dealt with in ASCA via the additive effect matrices, which are centered around the mean. In contrast, scaling is an important issue in ASCA. The possible effects on the analysis results are as large as in PCA, while the number of possible scaling strategies is much larger.

So far, in empirical applications, ASCA is predominantly applied to unscaled data (Jansen et al. 2005; Lemanska et al. 2012; Mazerolles et al. 2011; e.g., Smilde et al. 2005; Wang et al. 2009) and only incidentally to autoscaled data [i.e., each variable with length one across all observations (e.g., Ferreira et al. 2009)], or effect scaled data [i.e., separately scaled effect matrices (e.g., van Velzen et al., 2008)]. However, a rationale for the scaling applied is consistently lacking. This is a pity, since it hampers judging whether the solution interpreted optimally reflects the phenomena of interest.

In this paper, we aim at offering insight in which scaling strategies are rational in ASCA. We will show that scaling directly influences which data aspects are stressed in the analysis, and hence become apparent in the solution. We will argue that a proper scaling depends on which effects are of interest, and hence that different types of scaling may be proper for the different effect matrices. In the absence of an external objective criterion for judging the adequacy of the scaling strategies, we base our discussion of these strategies on theoretical justification and their impact on the solution.

We start by explaining, in mathematical terms, the ASCA model, its estimation, the effects of scaling on the solution and different scaling variants. For ease of explanation, we do so on the basis of a small experimental design that shows the basic principles relevant for scaling. The thus shown principles are generalizable to any, more complicated, experimental design. Next, we use a toy example to show the effects of various types of scaling on different effect matrices with a small simulated data set. Then, we analyze a real-life metabolomics data set. We end by discussing the implications when applying ASCA and related methods to scaled data in practice.

## Theory: ASCA model and scaling

### Model and estimation

For ease of explanation, we consider a small example: a nested balanced experimental design, with a between-subject factor ‘treatment’ and a within-subject factor ‘time’. There are *J* treatments (*j* = 1,…,*J*), with *I*_*j*_ = *I* subjects (*i*_*j*_ = 1,…,*I*) in treatment *j*. This yields *N* = *JI* subjects in total. Each subject is measured at *K* comparable time points (*k* = 1,…,*K*), implying that the scores at time point *k* can be sensibly compared across subjects (Van Mechelen and Smilde 2011). At each time point *k* for all *N* subjects, *L* dependent variables (e.g., metabolites) (*l* = 1,…,*L*) are measured. The observed scores can be collected in a data matrix **X** (*NK* × *L*).

The ANOVA model for a single metabolite *l*, can be formulated as (cf. Winer 1971)

1 $$x_{{jki_{j} }} = \mu + \alpha_{j} + \beta_{k} + \left( {\alpha \beta } \right)_{jk} + \varepsilon_{{jki_{j} }} ,$$

where *µ* indicates an overall offset, *α*_*j*_ the effect of ‘treatment’, *β*_*k*_ the effect of ‘time’, (*αβ*)_*kj*_ the interaction of ‘treatment’ and ‘time’, and $\varepsilon_{{jki_{j} }}$ the subject specific deviation. In ASCA, the data matrix **X** (*NK* × *L*) is first decomposed into a few effect matrices according to the model in Eq. (1), as:

2 $${\mathbf{X}} = 1 {\mathbf{m}}^{\text{T}} + {\mathbf{X}}_{\alpha } + {\mathbf{X}}_{\beta } + {\mathbf{X}}_{\alpha \beta } + {\mathbf{X}}_{\text{E}}$$

where **1** (*NK* × 1) consists of ones, **m** ^T^ (1 × *L*) contains the means of the *L* variables computed across all *NK* observations; **X**_*α*_ and **X**_*β*_ hold the level means for the factors ‘treatment’ and ‘time’, respectively; **X**_*αβ*_ the interaction terms for those two factors; and **X**_E_ the subject-specific effects. The latter express the variation between subjects at each time point within each treatment. Note that the effect matrices are highly structured: all rows related to one level of the factor in question are equal (e.g., all rows of **X**_*α*_ pertaining to treatment *j*). Further, all effect matrices have a sum constraint of zero to identify the ANOVA decomposition (e.g., $\sum\nolimits_{j = 1}^{J} {{\mathbf{x}}_{\alpha ,j}^{\text{T}} = {\mathbf{0}}^{\text{T}} } , \, \sum\nolimits_{k = 1}^{K} {{\mathbf{x}}_{\beta ,k}^{\text{T}} = {\mathbf{0}}^{\text{T}} }$, etc., where **x**_*α*,*j*_^T^ indicate the rows of effect matrix **X**_*α*_ pertaining to treatment *j*).

To identify the dominant sources of variation for the effects of interest, PCA is applied to the associated effect matrices or to an additive combination thereof. We consider two different types of effects that are of interest in practice, the between and within effects. Note that in most applications of ASCA only the between effect is considered, but, for example, Xu et al. (2014) study both.

The between effect expresses the main effect of treatment and its differential effects across time, and is modeled through a PCA of (**X**_*α*_ **+** **X**_*αβ*_) = **X**_(*α*+*αβ*)_.

The within effect pertains to the natural variation across individuals at each time point within each condition, and is analyzed via a PCA of **X**_E_. Herewith, one focuses on identifying variables with relatively large and similar residual patterns across time points and treatments. This may reveal important information on possible differential effects of a particular treatment condition across individuals. The size, onset and nature of treatment effects may substantially differ across individuals, even when they would be sampled from a (seemingly) single population. This opens the possibility of subtyping and understanding personalized treatment effects.

To understand the effects of scaling it is important to know that the separate PCA’s on the effect matrices are equivalent to the minimization of a specific least-squares estimation problem in terms of the observed data matrix **X**. For the model described above, the associated ordinary least-squares (OLS) loss function boils down to3where **T**_(.)_ (*NK* × *Q*_(.)_) and **P**_(.)_ (*L* × *Q*_(.)_) denote the component score matrix and loading matrix, respectively, for effect (.), with (*α* + *αβ*) the between effect and (E) the within effect; and subject to the sum constraint of zero for each effect matrix and each component score matrix (for details, see the Supplementary Material (SM)).

The fits of the different parts of an ASCA model are typically expressed as a percentage of variance explained (VAF). For the ANOVA part, we consider for each effect matrix the VAF of the total data (yielding the between-VAF and within-VAF). For the PCA parts, we consider for each component the VAF of its effect matrix. Regarding the number of components, we consider, in line with previous applications, maximally two components per effect matrix, retaining those components with a proper substantive interpretation. No formal criterion is available to indicate the number of components *Q*_(.)_ needed to adequately describe the between and within effects. Such a criterion could be based on principles used in other forms of component analysis (e.g., CHull (Ceulemans and Kiers 2006) or Parallel analysis (Horn 1965)), but their use in ASCA context is not straightforward.

In the ASCA model, the mean at each time point is explicitly modelled. This is possible because all subjects are measured at the very same time points. Explicit modeling of the mean is denoted as the fixed effects approach to modeling longitudinal data (Snijders and Bosker 1999). A more sophisticated alternative would be to model the repeated measures as if they are observations on a population of curves. Then, one could also model data collected at time points that vary across subjects and one would take the full advantage of any functional relationship between the time points. However, as combining such an approach with PCA is far from straightforward, this is topic of further research.

### Scaling in ASCA: weighting different parts

Scaling is defined as dividing each variable by a certain factor before subsequent analysis. Different scaling factors have been proposed, serving different goals, and with associated merits and drawbacks. For an overview we refer to van den Berg et al. (2006). Here, we consider the standard deviation, as a measure of data dispersion. Note, however, that the discussed consequences of scaling generalize to any other scaling factor considered.

Suppose that a scaled version ${\tilde{\mathbf{X}}}$ of the input data matrix **X** would be analyzed with ASCA. Specifically, assume that the data matrix was scaled by dividing each variable *l* by the corresponding standard deviation s_*l*_, *l* = 1,…,*L,* across all rows. This implies that ${\tilde{\mathbf{X}}} = {\mathbf{XW}}$ with ${\tilde{\mathbf{X}}}$ the scaled matrix, **X** the input data and **W** a diagonal weight matrix (*L* × *L*), with the inverse of the standard deviations s_*l*_, *l* = 1,…,*L*, on its diagonal. The consequences of this scaling for the estimated component score and loading matrices become clear by considering the OLS loss function (3), in terms of ${\tilde{\mathbf{X}}} = {\mathbf{XW}}$. As shown in the context of PCA and three-way component analysis, an OLS analysis of thus scaled data is equivalent to a weighted least squares (WLS) analysis of the input data (Bro and Smilde 2003; Harshman and Lundy 1984). For ASCA, starting from Eq. (3), this can be seen as follows:4with ${\tilde{\mathbf{m}}}^{\text{T}} = {\mathbf{m}}^{\text{T}} {\mathbf{W}}$, ${\tilde{\mathbf{X}}}_{\beta} = {\mathbf{X}}_{\beta } {\mathbf{W}}$, ${\tilde{\mathbf{X}}}_{{\left( {\alpha + \alpha \beta } \right)}} \varvec{ } = \, {\mathbf{X}}_{{\left( {\alpha + \alpha \beta } \right)}} {\mathbf{W}}_{{\left( {\alpha + \alpha \beta } \right)}}$, ${\tilde{\mathbf{X}}}_{\text{E}} = {\mathbf{X}}_{\text{E}} {\mathbf{W}}_{\text{E}}$, ${\tilde{\mathbf{P}}}_{{\left( {\alpha + \alpha \beta } \right)}} = {\mathbf{P}}_{{\left( {\alpha + \alpha \beta } \right)}} {\mathbf{W}}_{{\left( {\alpha + \alpha \beta } \right)}}$, ${\tilde{\mathbf{P}}}_{\text{E}} = {\mathbf{P}}_{\text{E}} {\mathbf{W}}_{\text{E}}$, and ${\mathbf{W}}_{{\left( {\alpha + \alpha \beta } \right)}} = {\mathbf{W}}_{\text{E}} = {\mathbf{W}}$. Thus, ASCA on the scaled matrix (i.e., ${\tilde{\mathbf{X}}} = {\mathbf{XW}}$) is equivalent to ASCA on the input data matrix (i.e., **X**) using a WLS loss function with weight matrix **W**. Note that this equivalence does hold because the weights are column specific (as, e.g., in autoscaling), and would not hold when the weights would be element specific (as in the general WLS definition, using the Hadamard product).

From Eq. (4), it follows that scaling boils down to differently weighting the variables when computing the solution and thus implies a change in the objective function, except for the trivial case of **W** = *c* **I**, with *c* an arbitrary constant. The ASCA solutions based on unscaled and scaled data will generally differ more with increasing variability in the diagonal elements of **W**. Generally, variables with relative large weights in **W** contribute to a larger extent to the loss value, and hence will influence the solution to a larger extent than in the unscaled analysis. In the unscaled data such variables will have small standard deviations, and thus are suppressed by the high-variance metabolites in the unscaled analysis.

Up to now, we focused on the case where the weight matrix is constant for each separate effect matrix. Yet, an obvious, and for ASCA possibly fruitful, alternative strategy is to use different weight matrices for each effect matrix under consideration. We will denote this type of scaling as effect scaling. In Eq. (4), effect scaling would boil down to taking ${\mathbf{W}}_{{\left( {\alpha + \alpha \beta } \right)}} \ne {\mathbf{W}}_{\text{E}}$. As a consequence, the thus obtained solutions are no longer equivalent to OLS-ASCA solutions of a scaled version of the input data matrix (i.e., of any ${\tilde{\mathbf{X}}} = {\mathbf{XW}}$). However, for each effect matrix separately, the OLS solution of the effect matrix (.) scaled with **W**_(.)_ is equivalent to a WLS solution of the unscaled effect matrix, with weight matrix **W**_(.)_. This is so because the scaling is still done per variable. This implies that—analogously to the effect of using a single weight matrix—the solutions based on unscaled and scaled data will generally differ more with increasing variability in the diagonal elements of **W**_(.)._

### Scaling in ASCA: choosing the weights

The key question now is how to select proper weight matrices for ASCA. A weight matrix is proper when the effects of interest are effectively displayed in the ASCA solution, despite of nuisance effects (i.e., effects that are not of interest). Thus, weights should be chosen such that the variability in the data due to nuisance effects is diminished, while leaving intact the variability due to the effect of interest.

In component analysis, it is rather common practice to use per variable (*l*) the observed standard deviation (sd_*l*_) as the scaling factor (i.e., *w*_*ll*_ = (sd_*l*_)^−1^). However, in ASCA, different types of standard deviations can be computed, with the differences pertaining to which conditions are considered and which effect matrices are used. Hence, the question is which of these standard deviations would most likely reflect only nuisance effects, and thus would be useful for scaling. Though the answer to this question partly depends on the study and data at hand, a general guideline can be given. The key is that the scaling factor should be free from the experimental effect of interest. A main effect of treatment, for example, shows up in variance across treatment levels. Using a scaling factor that includes the treatment variance thus results in downweighting variables with large treatment effects. Those variables may hence remain unnoticed. Because the effect of scaling depends on the mutual ratios in the weights that are used for the scaling, this undesirable effect can be expected to occur especially for variables for which the treatment effect makes up a relatively large part of the scaling factor. As we will explain, different effect matrices (i.e., between and within effect matrices) may require different types of scaling.

*Between effect* In decomposing ${\mathbf{X}}_{{\left( {\alpha + \alpha \beta } \right)}}$, one aims at identifying those variables for which the treatment effects and their differential effects across time are largest. A suitable scaling factor would be free from those sources of variance. We discuss three types of scaling factors, which all meet this requirement.

#### *1. Overall residual standard deviation*

The matrix **X**_E_ (*NK* × *L*) contains the residuals, and expresses the variation between subjects within each condition (i.e., time point and treatment combination). Using its columnwise sd (i.e., sd per column of **X**_E_) results in a downweighting of variables with relative large variation between subjects within conditions. This makes sense because for those variables the associated main and interaction effects will be estimated with less precision.

When the residual variations would differ largely across conditions for one or more variables, using the overall residual sd s as the scaling factor seems to be problematic, however. First, the size of an overall residual sd is influenced heavily by (some) large within-condition variances for a given variable. This may yield a disproportional downweighting of variables with small within-condition variances across some conditions. This undesirable effect would be precluded by taking a more robust measure, as the median of all within-condition variances per variable.

Second, a treatment that induces large effects in means across time, can very well be accompanied with increasing variability across subjects (Jansen et al. 2012). Then, the overall residual variance scaling would put less emphasis on those variables with large effects in means—which are to be identified. In this case, scaling with the overall residual sd is to be discouraged, and a measure expressing the amount of natural variation is needed.

#### *2. Natural residual variation: pre-intervention/reference group residual sd*

In case the experimental design includes a pre-intervention phase or a reference group, the natural variation within a condition can be estimated. When pre-intervention data are available, one could use the residual sd within all conditions using only the time point(s) before treatment actually starts (i.e., sd per column of ${\mathbf{X}}_{{{\text{E}}, {\text{pre}}}}$, with ${\mathbf{X}}_{{{\text{E}}, {\text{pre}}}}$ the part of **X**_E_ pertaining to the pre-intervention phase). If one of the treatment groups could be considered a reference group, pertaining to absence of treatment, or treatment as usual, one could to use the residual sd in the reference group (i.e., sd per column of ${\mathbf{X}}_{{{\text{E}}, {\text{ref}}}}$, with ${\mathbf{X}}_{{{\text{E}}, {\text{ref}}}}$ the part of **X**_E_ pertaining to the reference group, denoted as reference residual scaling for short). Indeed, for identifying a differential treatment effect, variables with relatively large variability in the reference group are of less interest than variables with a small variability.

#### *3. Reference group sd*

If there are variables with a substantial time effect (e.g., a trend) in the reference group, one may express this in the measure of natural variation. This can be done via the sd of the reference group scores on all time points (i.e., sd per column of **X**_ref_, with **X**_ref_ the observed scores in the reference group), to be denoted as reference group scaling for short. In this case one includes the main effect across time in the reference group in the scaling factor, implying that variables with a substantial time effect (e.g., a trend) are downweighted. Obviously, reference group scaling and reference residual scaling will only yield different results if a main effect across time is actually present in the reference group for at least one or the variables and this main effect differs considerably across variables.

*Within effect* Since analyzing the within effect aims at identifying differential effects of treatments on individuals, those effects should be excluded from the scaling factor. Further, a scaling factor including any between effect (e.g., a main effect across time in the reference group) appears strange, since the between effects themselves are of no influence on the residuals to be analyzed. However, it does make sense to correct for any differences in residual variability between variables in a reference condition. This suggests the use of—analogously to the between case—the pre-intervention or reference group residual sd. If both a pretest phase and reference condition are lacking in the experimental design, there seems no other option than to resort to no scaling.

The scaling factors based on residuals (i.e., of **X**_E_ or a submatrix thereof) requires the computation of **X**_E_. This is easily done by computing the centered version of each data matrix **X**_*jk*_ (*j* = 1,…,*J*; *k* = 1,…,*K*), containing the observed scores for treatment *j* at time point *k*, and positioning those matrices below each other.

## Simulated data example

### Simulated data

**Fig. 1:**
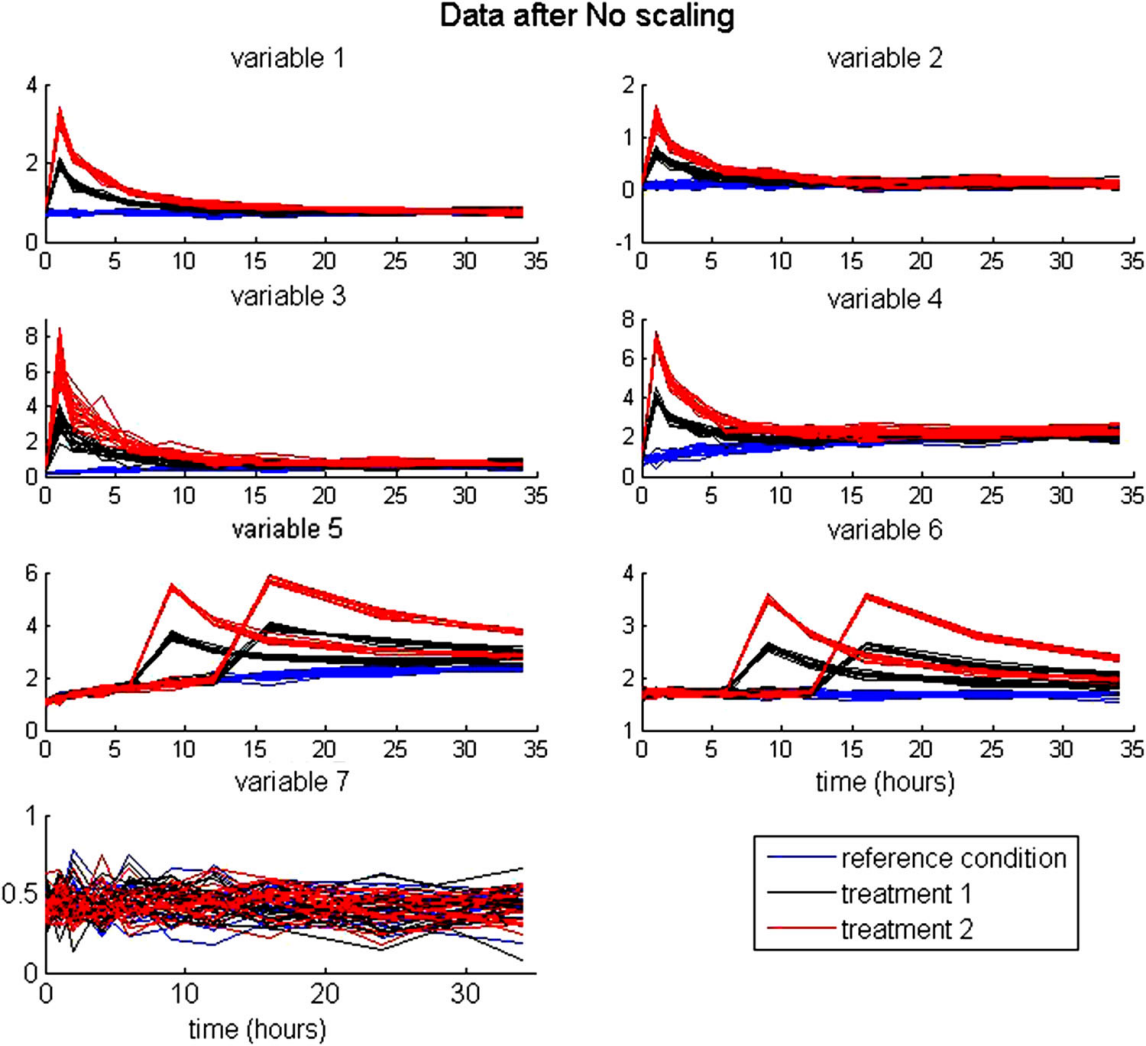
Simulated data, of 20 individuals per treatment condition, at 10 time points, on 7 variables

To illustrate the effects of suitable and less suitable scaling variants in ASCA, we use a small simulated data set. The data were simulated such that it comprises various effects, which can be identified with ASCA. The experimental design pertains to *J* = 3 treatments, including a reference condition (e.g., placebo), with per condition 20 individuals, who are measured at *K* = 10 non-equidistant time points, reflecting *t*_*k*_ = 0.00001,…,34 h after intake, on *L* = 7 variables. Details on the simulation model and parameters are provided in the SM. The simulated data are depicted in Fig. 1. Regarding between effects, expressing the treatments and their differential effects across time, there are three types of patterns. First, variables 1–4 (v1–v4 for short) have a very early treatment effect peak (i.e., at 1 h). Of those, v3 and v4 have a small and large, respectively, linear time trend, and v1 and v2 no trend. Second, v5 and v6 have a medium to late treatment effect peak; v5 has a linear trend, and v6 has no trend. Third, v7 shows no between effect. For all variables with a treatment effect (i.e., v1–v6), treatment 2 has larger effects than treatment 1, while the intensity of the effect differs between variables.

Regarding within effects, expressing interindividual differences across time and treatments, there are three types. For v1–v3, the variance of the residuals is proportional to the mean per time point and treatment condition. This property is visible best in Fig. 1 for v3 up to 5 h, since the associated means are higher than at later time points. For v5 and v6, two subtypes of individuals can be distinguished in the variables, namely those with a medium peak (around 9 h) and those with a late peak (around 18 h). For v4 and v7, the residuals are independent, with v4 having larger variances than v7.

### Between effects: analysis and results

For the between effects (i.e., related to (*α* + *αβ*)—see Eq. 3), we consider the effects of four scaling types. The types (and between-VAF) are no scaling (11.7 %), reference residual scaling (45.6 %), reference group scaling (47.8 %) and autoscaling (39.3 %), suggesting sufficient between-variability in the data to consider the between-components. The first three scaling types are suitable, but they stress different data aspects, though. Autoscaling, which involves as the scaling factor per variable the sd of the raw scores on the variable across all treatments, time points and individuals, is less suitable, since it includes treatment and differential treatment effects.

For each of the four between solutions, the first component is primarily related to v1 to v4, and the second to v5 and v6. Those two components cover the experimental effects present in those data. Because the effects of scaling can already be illustrated with the first component, we only discuss those results. Plots for the second component are provided in the SM (Fig. 1).

**Fig. 2:**
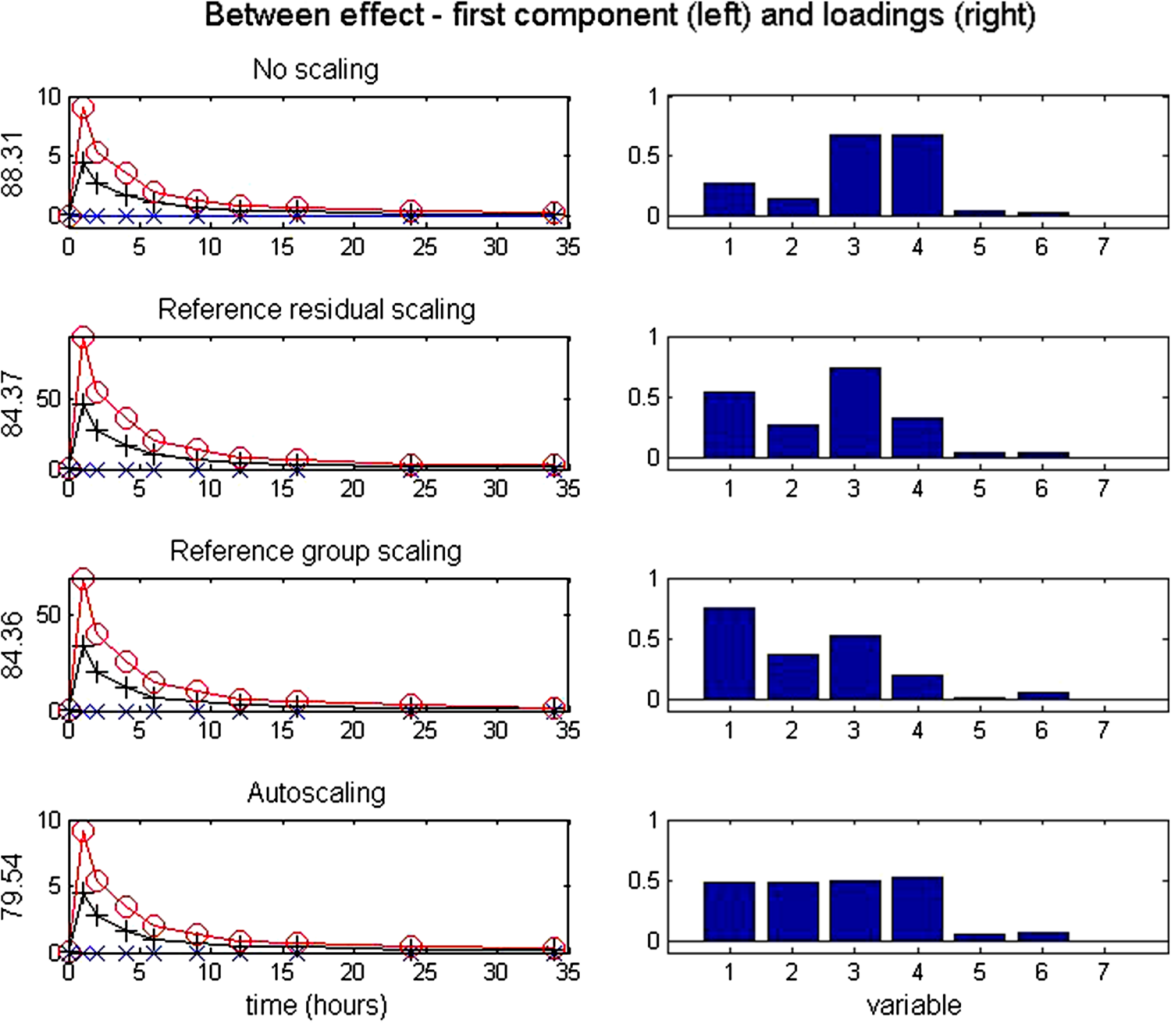
Simulated data: between effect after four scaling types. *Left* scores on the first component across time for each treatment condition, with the VAF of the between effect matrix; *right* the associated loadings

Figure 2 depicts per scaling type, the scores on the first component across time per condition (left), and the associated loadings (right). As can be seen in the left part of Fig. 2, the component scores show highly similar patterns across time for each type of scaling. This implies that the associated loadings can be mutually compared directly. The loadings of v1–v4 show clear differences in the relative importance of the variables. The loadings of no scaling express the relative effect sizes—which can be seen already in Fig. 1—with relative large sizes for v3 and v4, medium for v1 and small for v2.

The loadings of reference residual scaling express the effect sizes of treatment relative to the residual variance within the reference condition. The loading for v1 is largest, followed by v3, v4, and v2. As can be verified in Fig. 1, this ordering of effects indeed reflect the balance between (relative larger) treatment effects and (relative smaller) residual variances. That is, v1 has a low peak, but very low residual variance in the reference condition; v3 and v4 both have a high, and about equal, peak, and relatively low, and about equal reference residual variances. Finally, v2 has a low peak, with relatively large residual variance.

The loadings of the reference group scaling express the treatment effect sizes relative to the total variance within the reference condition. When comparing this scaling type to reference residual scaling, it is to be expected that v3–v5 will be downweighted, because v3–v5 have a trend across time, and the other variables have no trend. The trend for v3 being smaller than for v4 and v5, results in less downweighting of v3. Comparing the obtained relative loadings with those after reference residual scaling shows indeed lower loadings for v3–v5, with the largest decrease for v4 and v5, as expected.

The loadings of the autoscaling express the treatment effect sizes relative to a contstant, namely the overall variance of each variable. For v1–v4 this ratio is about equal, as indicated by the associated loadings for v1–v4. This implies that autoscaling fails to reflect the relative sizes of the treatment effect.

### Within effects: analysis and results

**Fig. 3:**
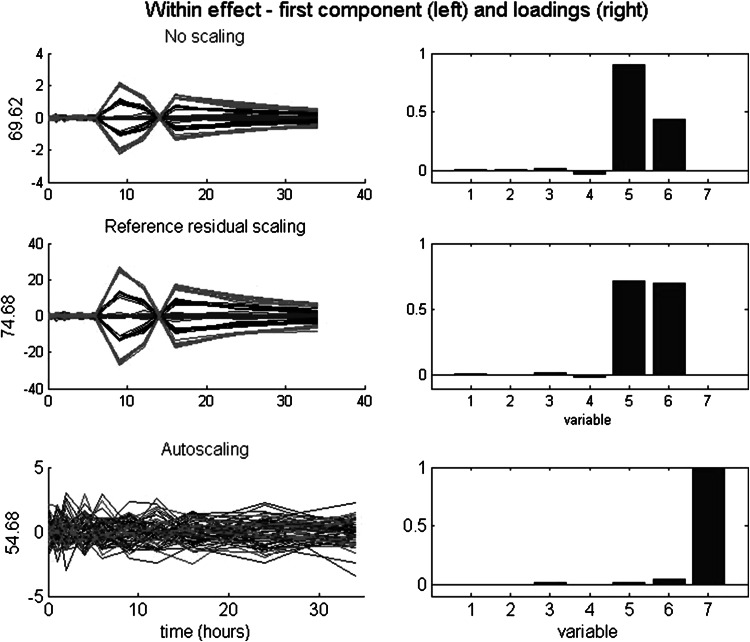
Simulated data: within effect after three scaling types. *Left* scores on the first component across time per condition, with the VAF of the within effect matrix; *right* the associated loadings

For the within effects (i.e., related to (E)—see Eq. 3), we consider the effects of three scaling types. The types (and within-VAF) are no scaling (2.4 %), reference residual scaling (11.6 %), and autoscaling (24.6 %). Only the first two are suitable scaling types. Figure 3 depicts the scores on the first within component across time per condition (left) and the associated loadings (right) per scaling type. As can be seen in Fig. 3, the pattern in scores for no scaling and reference residual scaling expresses the interindividual differences in treatment response across time. The plot clearly shows two subtypes in responders which are present for v5 and v6. The key difference is in the estimated relative size. For v5, the double peaks, and thus the associated individual deviations from the mean, are higher than for v6. The loadings of no scaling express this absolute difference. In contrast, the loadings of the reference residual scaling express the balance between (large) individual deviations and (small) reference residual variance. This balance apparently is about equal for v5 and v6. Autoscaling equalizes the total variance across variables. Because v7, unlike v1–v6, has no between variance, its within variance is massive in comparison to the remaining variables. Therefore, v7 dominates the first within component after autoscaling, showing the—uninteresting—independent residuals across time. The two subtypes in responders are visible in the second within component after autoscaling (not shown).

## Empirical data example: a nutrikinetics study

### Study design

To study the bioavailability of polyphenols, 20 male subjects underwent a treatment with a tea extract, a wine extract or a placebo extract in a cross-over design. Blood samples were collected just before (0 h) and at 1, 2, 6, 9, 12, 24 and 36 h after the intake. The chemical identities of the set of (poly-)phenolics were known prior to the start of the study (i.e., targeted analysis). Pure standards were used for the identification and quantification (see Materials and Methods in van Velzen et al. 2014). The resulting data set consists of measurements of 11 metabolites. From those 11 metabolites, two are deleted from the data because there are so many zeroes in the data that the variances of the different groups could not be determined. We refer to v1–v9 to indicate the metabolites; their names are listed in the SM. There is large interindividual variation which is often the case in nutritional studies. To illustrate the effects of scaling in ASCA, the data are now subjected to different types of scaling before the ASCA analysis, among which scaling with a reference group (i.e., the placebo group). Note that scaling using the pretest data (i.e., at 0 h) would be possible as well, but adds little to the illustration, since the residual variances at pretest are rather similar to the residual variances in the placebo group.

Of the raw data (depicted in the SM, Fig. 2), the total variances differ greatly across metabolites. The metabolite v7 has by far the largest variance (s^2^ = 682.9), followed by v3 (s^2^ = 160.5). The four lowest variance metabolites have a maximum variance of only s^2^ = 2.6 (v1, v5, v6 and v8).

After reference residual scaling (depicted in the SM, Fig. 3), the situation is drastically changed compared to the unscaled data. In the reference group scaled data, v8 has the largest variance (s^2^ = 169.5), followed by v2 (s^2^ = 73.7). The top two largest variance metabolites of the raw data (v7 and v3) are at the 3^rd^ and 6^th^ position (with s^2^ = 42.3 and 5.8, respectively) after reference residual scaling. As we verified, but do not show, the reference group scaled data closely resembles the data after reference residual scaling due to lack of a time trend of the placebo treatment for all variables involved.

With respect to the time profiles, v3, v4 and v7 show high resemblances. In the time profile of these three metabolites shortly after the start of the experiment the concentration starts to rise, the most for tea, and the maximum is reached after approximately 2 h after which the decrease starts. Further, v8 and v9 have maximum values at around 10 h, which is much later than the peaks for v1–v7.

### Between effects: analysis and results

**Fig. 4:**
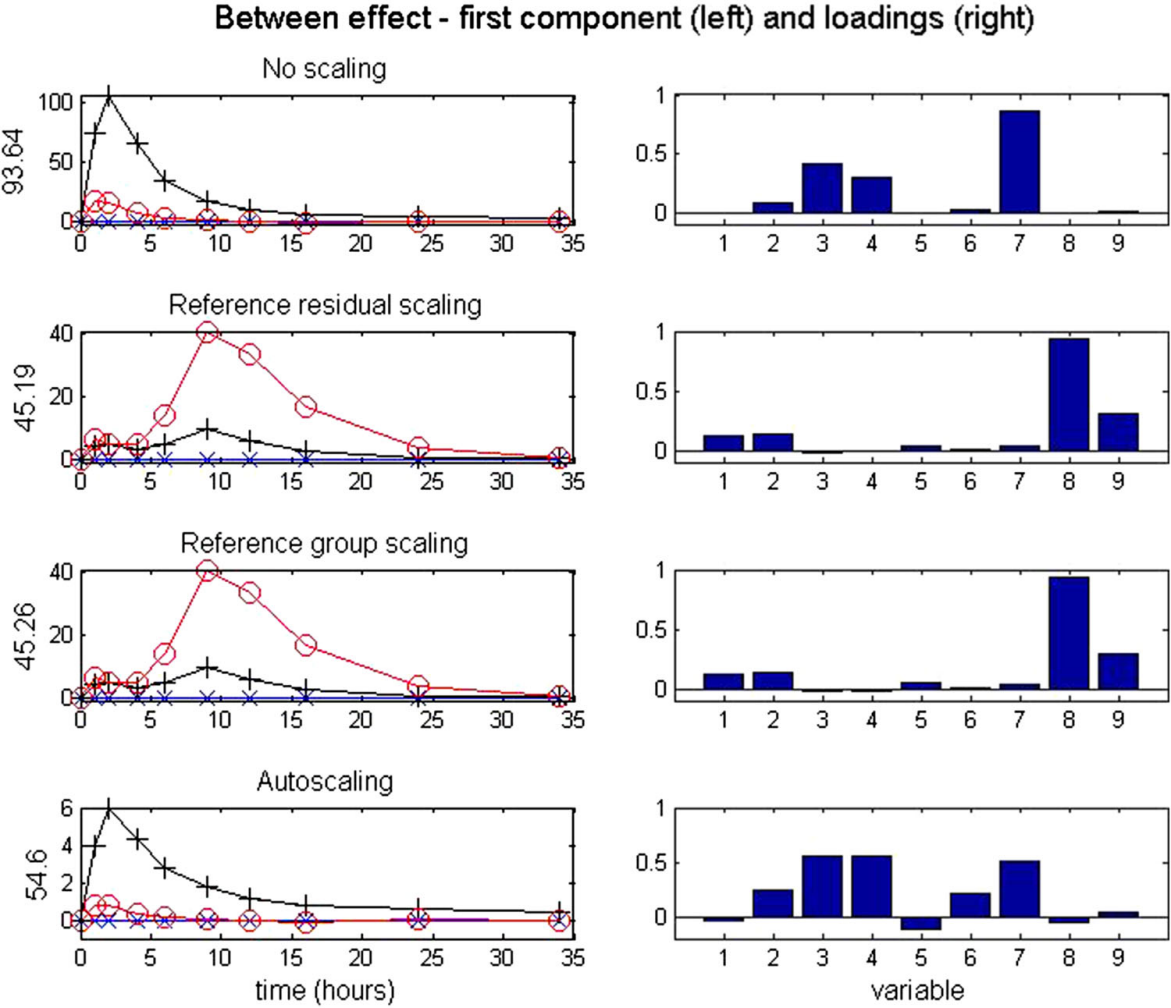
Empirical data: between effect after four scaling types. *Left* scores on the first component across time per condition, with the VAF of the between effect matrix; *right* the associated loadings

Analogously to the analyses of the simulated data, we consider the between effects (with between-VAF) after no scaling (36.7 %), reference residual scaling (32.4 %), reference group scaling (32.4 %), and autoscaling (33.7 %). Figure 4 depicts the scores on the first component across time per condition (left) and the associated loadings (right). In Fig. 4, the component scores of the no scaling case show a fast response after the start of the experiment with a maximum at 2 h, after which a decay starts. As can be seen from the associated loadings, the first component in no scaling is primarily determined by v7, and to a somewhat lesser extent v3 and v4. Their profiles resemble the profile of v7, albeit at a lower height. This result is in agreement with the data.

Applying reference residual scaling and reference group scaling yields very similar data and therefore also the ASCA results closely resemble one another. As can be seen in Fig. 4, v8 and to a lesser extent v9 are the important metabolites. Those two metabolites have their maximum value around 10 h after which there is a decrease. This is in agreement with the data after reference group scaling.

For the between effect, autoscaling and no scaling yield similar results in that the same variables (v3, v4, v7) become apparent as important. However, the associated loadings indicate similar relative importance for those variables, which obviously was not the case in the no scaling solution.

For this dataset the scaling type has a large influence on the first component of the between effect. That is, the first between components after no scaling and autoscaling show totally different aspects of the data when compared to the first between component after reference condition based scalings. This suggests that a single component is insufficient to adequately describe the data, and therefore it was decided to have a look at the second component of the between effect.

**Fig. 5:**
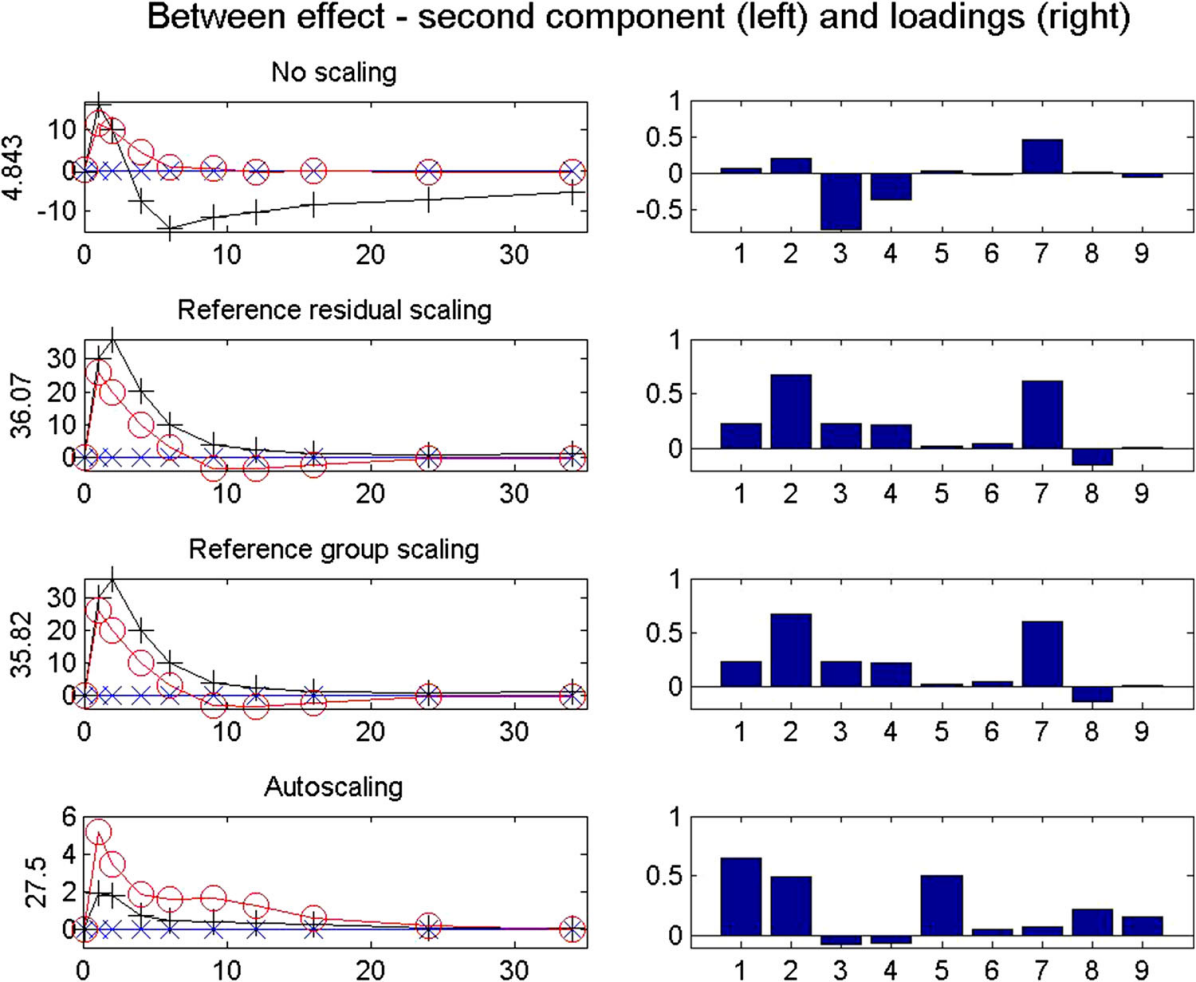
Empirical data: between effect after four scaling types. *Left* scores on the second component across time per condition, with the VAF of the between effect matrix; *right* the associated loadings

Figure 5 depicts the second between component after the four scaling types. In this figure, it can be observed that in the second component for the unscaled data the variables with the early peak still dominate. The signs in loadings (positive for v7 and negative for v3 and v4), combined with the component scores with a maximum at 2 h and a minimum at 6 h, reflect that v7 shows a much faster decay than v3 and v4. The variance in those three metabolites is so dominant that the other metabolites (i.e., those with a peak around 10 h, or with a larger effect for wine than for tea) hardly play a role in the first two components. With reference residual scaling and reference group scaling, the second component expresses the early peak effect, largest for tea and followed by wine, primarily for v2 and v7. With autoscaling, also an early peak effect is expressed, but now with the largest effect for wine, primarily for v1, v2 and v5.

### Within effects: analysis and results

**Fig. 6:**
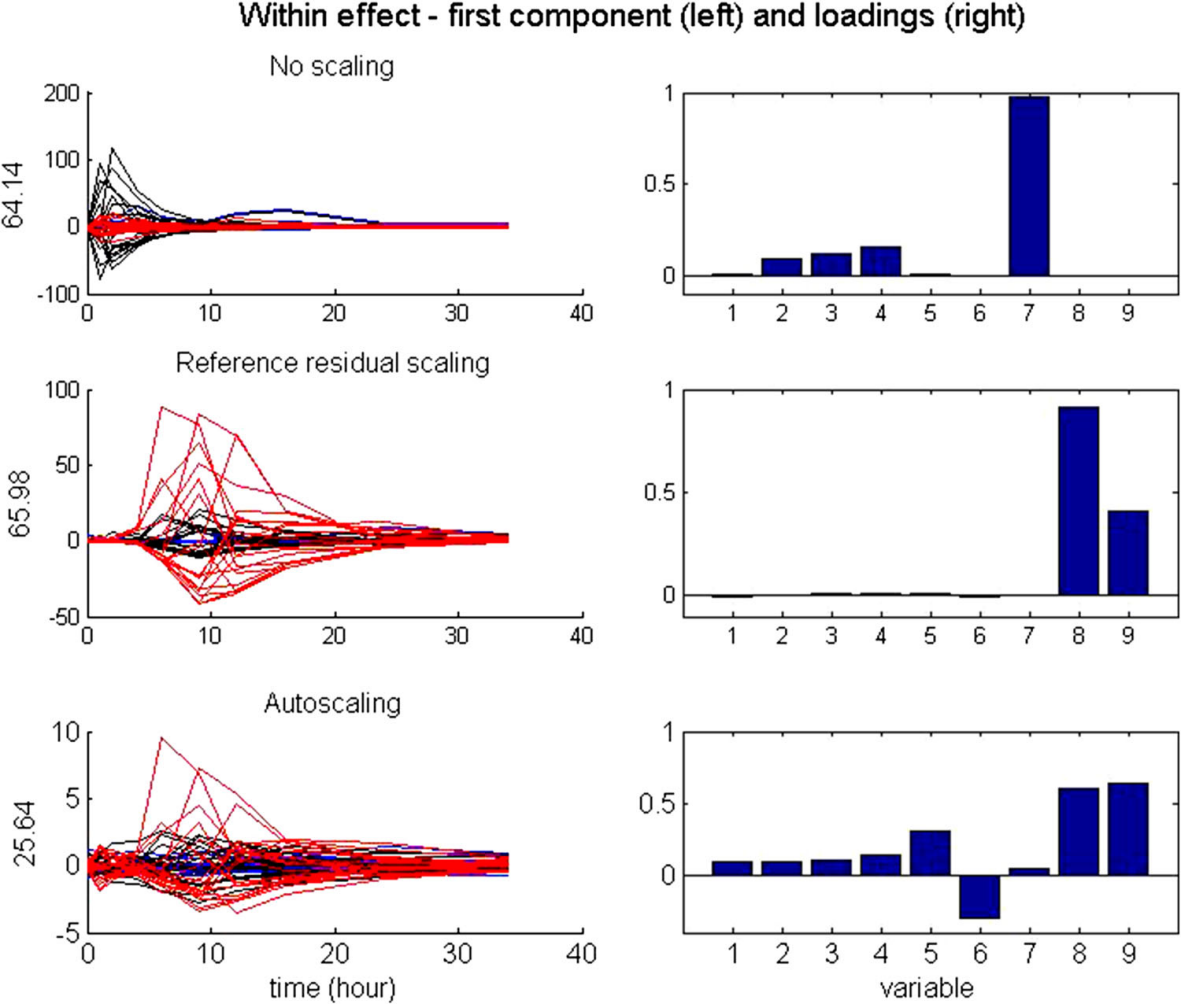
Empirical data: within effect after three scaling types. *Left* scores on the first component across time per condition, with the VAF of the within effect matrix; *right* the associated loadings

We considered the within-effects after no scaling (within-VAF 22.0 %), reference residual scaling (45.0 %) and autoscaling (48.3 %). In Fig. 6, the scores on the first component are plotted across time for each condition (left), and the associated loadings (right) after no scaling, reference residual scaling and autoscaling. As can be seen in this figure, the within effect after no scaling results in a high importance of variable 7. This reflects the relatively large within variance in the tea condition, combined with the fact that this metabolite has by far the largest values in the data.

Both after autoscaling and reference residual scaling, the first component of the within effect describes the individual differences for v8 and v9. In the raw data it appears that the maxima for v8 and v9 differ between the different individuals. This phenomenon is picked up by autoscaling and reference residual scaling.

## Concluding remarks

ASCA is powerful for identifying sources of variance due to experimental manipulations among large numbers of variables. We showed the importance of proper scaling in ASCA. Proper scaling uses scaling factors free from the effect of interest, thus excludes effects induced by the experimental manipulation. Therefore, autoscaling is to be discouraged, as it covers between-treatment variances. For designs involving a reference group, we advise considering the reference group sd as a scaling factor. For within effects, the residual variance is appropriate, while for between effects, the total variance (i.e., including any trend) could also be useful. Further, we advise to make and interpret plots of the unscaled and scaled data per variable—to gain insight into the effects of the scaling applied.

The reference group sd may be (close to) zero for particular variable(s), for example, because consistently small values are obtained. This would yield undefined (or extremely large) scaled variable(s); as a work-around one could exclude the variable(s) concerned, analyze unscaled data, or use as the scaling factor the standard deviation plus a threshold as in Tusher et al. (2001).

The issue of scaling is also highly relevant to other advanced methods based on separating sources of variation according to the experimental design. One can think of classification methods, as support vector machines and random forests (of which the large effects of scaling have been recently illustrated (Gromski et al. 2014), and of methods intimately related to ASCA (Smilde et al. 2012), as Scaled-to-maximum, aligned, and reduced trajectories (Keun et al. 2004).

## Electronic supplementary material

Below is the link to the electronic supplementary material.

Supplementary material 1 (DOCX 104 kb)
