# Supplementary material for: Scaling in ANOVA-simultaneous component analysis
Source: Metabolomics. 2015 Feb 14;11(5):1265–76. doi: 10.1007/s11306-015-0785-8 (PMC4559107; doi:10.1007/s11306-015-0785-8)
Supplement: Supplementary file 1 — Supplementary material 1 (DOCX 104 kb) [file 11306_2015_785_MOESM1_ESM.docx]

Supplementary material ‘Scaling in ANOVA-simultaneous component analysis’

# Separate PCA’s on effect matrices equal a least-squares problem on the observed data matrix

The separate PCA’s on the effect matrices can be viewed as a least-squares estimation problem, in terms of the observed data matrix **X** and sum constraints of zero on each of the component matrices. This is so because all effect matrices (i.e., $\boldsymbol{1} \mathbf{m}^{T}$, **X***_α_*, **X***_β_* **X***_αβ_*, $\mathbf{E}$ in Equation (2)) are mutually orthogonal, and because the PCA on the effect matrices results in component matrices that satisfy the sum constraints (Jansen et al., 2005; Timmerman, 2006). For the ASCA model involving the between and within effects, the associated ordinary least-squares (OLS) loss function boils down to

| ${}_{\mathbf{T}_{\left( \alpha+\alpha\beta\right)}\boldsymbol{,}\mathbf{P}_{\left( \alpha+\alpha\beta\right)}\boldsymbol{,}\mathbf{T}_{E}\boldsymbol{,}\mathbf{P}_{E}}^{\mathrm{argmin}}{\left\Vert\mathbf{X-1}\mathbf{m}^{T}- \mathbf{X} \mathbf{-}\mathbf{T}_{\left( \alpha+\alpha\beta\right)}\mathbf{P}_{\left( \alpha+\alpha\beta\right)}^{T}-\mathbf{T}_{E}\mathbf{P}_{E}^{T} \right\Vert^{2}=}$  ${}_{\mathbf{T}_{\left( \alpha+\alpha\beta\right)}\boldsymbol{,}\mathbf{P}_{\left( \alpha+\alpha\beta\right)}\boldsymbol{,}\mathbf{T}_{E}\boldsymbol{,}\mathbf{P}_{E}}^{\mathrm{argmin}}{\left\Vert\mathbf{X-1}\mathbf{m}^{T} \right\Vert^{2}+\left\Vert\mathbf{X}- \mathbf{X} \right\Vert^{2}+\left\Vert\mathbf{X-}\mathbf{T}_{\left( \alpha+\alpha\beta\right)}\mathbf{P}_{\left( \alpha+\alpha\beta\right)}^{T} \right\Vert^{2}+ \left\Vert\mathbf{X-}\mathbf{T}_{E}\mathbf{P}_{E}^{T} \right\Vert^{2}}$=  ${}_{\mathbf{T}_{\left( \alpha+\alpha\beta\right)}\boldsymbol{,}\mathbf{P}_{\left( \alpha+\alpha\beta\right)}\boldsymbol{,}\mathbf{T}_{E}\boldsymbol{,}\mathbf{P}_{E}}^{\mathrm{argmin}}{\left\Vert\mathbf{X}_{\left( \alpha+\alpha\beta\right)} \mathbf{-}\mathbf{T}_{\left( \alpha+\alpha\beta\right)}\mathbf{P}_{\left( \alpha+\alpha\beta\right)}^{T} \right\Vert^{2}+\left\Vert\mathbf{X}_{E}-\mathbf{T}_{E}\mathbf{P}_{E}^{T} \right\Vert^{2}},$ |  |  |
| --- | --- | --- |

where **T**_(.)_ (*NK ×* *Q*_(.)_) and **P**_(.)_ (*L × Q*_(.)_) denote the component score and loading matrices for effect (.), with (*α* + *αβ*) the between effect and (E) the within effect; further there are sum constraints of zero on each effect matrix and each component score matrix (i.e., $\sum_{k=1}^{K} \mathbf{x}_{\beta,k}^{T}$=**0**^T^; $\sum_{j=1}^{J} \mathbf{x}_{(\alpha+\alpha\beta),jk}^{T}=\mathbf{0}^{T}\boldsymbol{, \forall}k; \sum_{k=1}^{K} \mathbf{x}_{\left( \alpha+\alpha\beta\right),jk}^{T}=\mathbf{0}^{T}\boldsymbol{, \forall}j;$ $\sum_{i=1}^{I} \mathbf{x}_{\mathbf{E}\mathbf{,}jki_{j}}^{T}=\mathbf{0}^{T}\boldsymbol{, \forall}j,k$; $\sum_{j=1}^{J} \mathbf{t}_{(\alpha+\alpha\beta),jk}^{T}=\mathbf{0}^{T}\boldsymbol{, \forall}k; \sum_{k=1}^{K} \mathbf{t}_{(\alpha+\alpha\beta),jk}^{T}=\mathbf{0}^{T}\boldsymbol{, \forall}j$; $\sum_{i=1}^{I} \mathbf{t}_{\mathbf{E}\mathbf{,}jki_{j}}^{T}=\mathbf{0}^{T}\boldsymbol{, \forall}j,k$. The constraints on the component matrices are active in the first and second terms of Equation 5, but inactive in the third term, implying that the PCA’s of the effect matrices result in component matrices that meet the required constraint.

# Generation of simulated data

The experimental design pertains to 3 treatments (*j*=1,…,*J*), including a reference condition (*j =* 1), with per condition *I =* 20 individuals (*i*=1,…,*I*), who are measured at 10 non-equidistant time points (*k =* 1,…, *K*), on 7 variables (*l*=1,…,*L)*. The *k =* 1,…, *K* time points reflect *t_k_ =* 0.00001, 1, 2, 4, 6, 9, 12, 16, 24, and 34 hours after intake, respectively.

The simulated scores were based on three basis functions, C1*_k_*, C2*_k_* and C3*_k_*, evaluated at the 10 time points *t_k_*. Those functions express an early, middle and late peak, respectively, and they were generated as:

C1*_k_*= k1 * (exp(-k1 * *t_k-1_*) - exp(-k2 * *t_k-1_*)) / (k2 - k1),

C2*_k_* = k1 * (exp(-k1 * *t_k-5_*) - exp(-k2 * *t_k-5_*)) / (k2 - k1),

C3*_k_* = k1 * (exp(-k1 * *t_k-7_*) - exp(-k2 * *t_k-7_*)) / (k2 - k1),

with t*_k-x_* = 0 for *x* > *k*, k1 = 50 and k2 = 100.

The simulated scores V*l_ijk_* (on the *l*^th^ variable, for individual *i* in treatment condition *j* at time point *k*) were generated as follows:

V1*_ijk_* = {abs(rn*_l_*(0,1) + (*j −* 1) * 4 * C1*_k_*) .* (1+ rn*_ijk_*(0,.05)} + rn*_ijk_*(0,.03333)

V2*_ijk_* = {abs(rn*_l_*(0,1) + (*j −* 1) * 2 * C1*_k_*) .* (1+ rn*_ijk_*(0,.1)} + rn*_ijk_*(0,.05)

V3*_ijk_* = abs(rn*_l_*(0,1) + (*j −* 1) * 10 * C1*_k_* + 0.05**k*) * (1+ rn*_ijk_*(0,.2)

V4*_ijk_* = abs(rn*_l_*(0,1) + (*j −* 1) * 10 * C1*_k_* + 0.15**k*) + (1+ rn*_ijk_*(0,.2))

V5*_ijk_* = abs(rn*_l_*(0,1)) + 0.15 * *k* + (*j −* 1) * 3* C2*_k_* + rn*_ijk_*(0,.1), for *i* = 1,…,10

V5*_ijk_* = abs(rn*_l_*(0,1) + 0.15 * *k* + (*j −* 1) * 3*C3*_k_* + rn*_ijk_*(0,.1), for *i* = 11,…,20

V6*_ijk_* = abs(rn*_l_*(0,1) + (*j −* 1) * 3 * C2*_k_* + rn*_ijk_*(0,.05), for *i* = 1,…,10

V6*_ijk_* = abs(rn*_l_*(0,1) + (*j −* 1) * 3*C3*_k_* + rn*_ijk_*(0,.05), for *i* = 11,…,20

V7 *_ijk_* = abs(rn*_l_*(0,1) + rn*_ijk_*(0,.1),

where rn*_._*(0,1) is a randomly drawn number from *N*(0,1), and abs(.) indicates the absolute value.

# Additional figures

****Figure 1 Between effect after four types of scaling of simulated data. Left: scores on the second component plotted across time for each condition; right: the associated loadings for v1 to v7

Figure 2 Measured nutrikinetics data, of 20 individuals per treatment condition, at 8 time points, on 9 variables

Figure 3. The nutrikinetics data after reference residual scaling

# List of metabolites used in Empirical data example (nutrikinetics study)

The metabolites, using names according to the Pubchem ontology, and their abbreviations in the text are as follows.

Metabolite Abbreviation in text

(-)-Catechin v1

(-)-Epicatechin v2

(-)-Epicatechin gallate v3

(-)-Epigallocatechin gallate v4

Resveratrol v5

Isorhamnetin v6

3/4-O-methylgallic acid v7

5-(3′-Methoxy-4′-hydroxyphenyl)-gamma-valerolactone v8

5-(3′,4′-Dihydroxyphenyl)-gamma-valerolactone v9
